# *Anaplasma* Species in Africa—A Century of Discovery: A Review on Molecular Epidemiology, Genetic Diversity, and Control

**DOI:** 10.3390/pathogens12050702

**Published:** 2023-05-12

**Authors:** Agatha Kolo

**Affiliations:** Department of Molecular Microbiology and Immunology, The University of Texas at San Antonio, San Antonio, TX 78249, USA; agatha.kolo@utsa.edu; Tel.: +1-210-296-4256

**Keywords:** *Anaplasma* spp., molecular epidemiology, genetic diversity, various hosts, ticks, control, Africa

## Abstract

*Anaplasma* species, belonging to the family Anaplasmataceae in the order Rickettsiales, are obligate intracellular bacteria responsible for various tick-borne diseases of veterinary and human significance worldwide. With advancements in molecular techniques, seven formal species of *Anaplasma* and numerous unclassified species have been described. In Africa, several *Anaplasma* species and strains have been identified in different animals and tick species. This review aims to provide an overview of the current understanding of the molecular epidemiology and genetic diversity of classified and unclassified *Anaplasma* species detected in animals and ticks across Africa. The review also covers control measures that have been taken to prevent anaplasmosis transmission on the continent. This information is critical when developing anaplasmosis management and control programs in Africa.

## 1. Introduction

The *Anaplasma* genus was discovered over a century ago in 1910 by Sir Arnold Theiler in South Africa [1,2]. *Anaplasma* spp. are the causative agent of the tick-borne disease anaplasmosis, which has a significant impact on animal and human health worldwide [3,4]. Currently, the genus *Anaplasma* has seven formally described species: *A. marginale*, *A. centrale*, *A. ovis*, *A. bovis*, *A. phagocytophilum*, *A. platys* and *A. caudatum* [5]. In addition to the classified *Anaplasma* species, the literature proposes the existence of additional *Anaplasma* species, including *Anaplasma odocoilei*, *Anaplasma* sp. Omatjenne, and *Anaplasma capra*. However, these potential species have not yet been formally described [6,7]. In sub-Saharan Africa, anaplasmosis is generally regarded as one of the important tick-borne diseases (TBD) of livestock causing significant economic losses to farmers in endemic areas [8]. Over the last decade, the use of molecular techniques has allowed the detection of *A. marginale*, *A. centrale*, *A. phagocytophilum*, *A. platys*, *A. bovis* and *A. ovis* in various animals and tick species across the length and breadth of the continent [8,9,10,11,12,13,14,15,16,17,18].

Since the last taxonomic reclassification over two decades ago [5], many unclassified species of *Anaplasma* have been recorded in the literature that are known or assumed to be tick-borne. Several of these putative *Anaplasma* species have been detected and reported in Africa. These include *Anaplasma* sp. SA dog from dogs in South Africa and Zambia [14,18,19,20], *Candidatus* Anaplasma boleense and *Anaplasma* sp. Mymensingh from cattle in South Africa [14], *Anaplasma* sp. Omatjenne from goats and cattle in South Africa, Ethiopia, Côte d’Ivoire, Zambia, Rwanda and Morocco [6,21,22], *Candidatus* Anaplasma ivoriensis from *Amblyomma* and *Rhipicephalus* ticks in Cote d’Ivoire [23] and *A. capra* detected from cattle in Angola [24]. Other novel *Anaplasma* spp. detected in Africa include *Anaplasma* sp. G75 from *Ixodes* ticks in Ghana [25], *Candidatus* Anaplasma camelii in camels, *Hyalomma*, *Amblyomma* and *Rhipicephalus* ticks from Nigeria and Kenya [26,27], *Candidatus* Anaplasma africae from sheep, goats and cattle in Senegal [28], *Anaplasma* sp. Hadesa from cattle in Cameroon and Ethiopia [29,30], *Anaplasma* sp. Saso from cattle in Ethiopia [30], *Anaplasma* sp. Lambwe from cattle in Kenya [16] and *Candidatus* Anaplasma sphenisci from African penguins in South Africa [31].

It is noteworthy that many of these studies used the 16S ribosomal RNA gene to detect these novel *Anaplasma* species. *Anaplasma* 16S rRNA gene sequences are very similar, often with identity scores >98%; therefore, discretion must be used when designating species in the genus [32]. The use of several genetic markers is also necessary to provide sufficient delineation of the different *Anaplasma* species [32].

Wildlife including wild ungulates and rodents are suggested to play a role in the epidemiology of anaplasmosis [33]. However, there is still a scarcity of information on the role wildlife play as reservoirs of *Anaplasma* spp. in Africa. It is therefore important to investigate *Anaplasma* infections in wildlife and their vector ticks in different regions to better understand the eco-epidemiology of anaplasmosis on the continent.

Some reviews have been done on the epidemiology and genetic diversity of *Anaplasma* spp. in Africa [34,35]; however, they were largely centered on regional studies. This is the first review to comprehensively delve into the body of work done on the molecular epidemiology and control of *Anaplasma* spp. with a spotlight on the entire continent. This review is focused on the current knowledge on the molecular epidemiology and genetic diversity of classified and unclassified *Anaplasma* spp. detected in various animals and ticks across Africa and the control of anaplasmosis in Africa. Data on the molecular epidemiology and genetic diversity of *Anaplasmataceae* around Africa in ticks and various hosts that includes the formally described species as well as putative and novel *Anaplasma* species are presented in the first section. In the second section, contemporary measures that have been developed to control the disease on the continent are presented. Final remarks on future research that could unveil the complete diversity of *Anaplasmataceae* and overcome some of the current challenges of *Anaplasma* taxonomy in Africa are subsequently presented. For the purposes of the study, the following index terms were searched for in PubMed, ScienceDirect, Google Scholar, Web of Science and Springer Link databases: “*Anaplasma*”, “Anaplasmataceae”, “molecular detection”, “molecular characterization”, “genetic diversity”, and “prevalence” in association with “ticks”, “cattle”, “dogs”, “sheep”, “goats”, “livestock”, “humans”, “wildlife” and “Africa”.

## 2. Molecular Epidemiology and Genetic Diversity of *Anaplasma* Species in Africa

### 2.1. Anaplasma Marginale

Bovine anaplasmosis is an important tick-borne rickettsial disease responsible for significant economic losses in the livestock industry worldwide [4]. The disease is caused by *A. marginale* and to a lesser extent *A. centrale*. *A. marginale* is biologically transmitted by nearly 20 tick species and is the most prevalent tick-borne pathogen globally [4]. Wild ruminants including buffalo, Rocky Mountain elk, wildebeest, black-tailed deer, white-tailed deer, mule deer and American bison have been largely regarded as reservoir hosts of *A. marginale* infection [36,37,38]. The disease is more severe in animals older than two years and causes a milder infection in younger animals. Clinical signs of infection include inappetence, weight loss, jaundice, reduced meat and milk production and possible death [4]. Control measures of bovine anaplasmosis typically involve the use of chemical acaricides to control the tick vector and the use of long-acting antibiotics such as oxytetracycline [39]. Genetic markers used for the characterization of *A. marginale* strains in Africa include the major surface proteins *msp1α*, *msp1β*, *msp4*, *msp5*, heat-shock protein (*groEL*), *dnaA*, *ftsZ*, *recA*, *secY*, *lipA*, *sucB*, *OmpA*, 23S ribosomal ribonucleic (rRNA) and 16S rRNA genes [12,13,16,21,23,29,40,41,42,43,44,45,46,47,48,49,50,51,52,53,54,55,56,57] (Table 1).

In southern Africa, specifically South Africa, *A. marginale* infection in cattle is endemic across the cattle farming regions of the country [39,58,59,60,61]. A survey of ticks collected from cattle and sheep across three provinces detected *A. marginale* in 3.8% of *Rhipicephalus decolaratus* ticks using *msp5* gene PCR and sequencing [12]. Characterization of *A. marginale* genotypes in blood samples collected from African buffalo, waterbuck, eland, black wildebeest, blue wildebeest and cattle using the 16S rRNA, *groEL* and *msp4* genes found two *A. marginale* genotypes of each gene circulating in the animals [62]. Recent research investigating the infection dynamics of *A. marginale* in 10 calves in two habitat areas at a wildlife–livestock interface in the country identified over 50 *A. marginale msp1α* genotypes and five novel *msp1*a repeats reveling in the calves over a 12-month period [51].

In Mozambique, 97 African buffalo were screened for *Anaplasma* species using quantitative PCR (qPCR) assays targeting the *msp1β* gene of *A. marginale*, with 72.2% of samples positive for *A. marginale* [50]. Positive samples were then sequenced using the *msp5*, *groEL* and 16S rRNA genes. Phylogenetic analysis revealed that *A. marginale msp5* gene sequences were clearly separated from *A. centrale* sequences by a genetic divergence of 14%. Sequence analysis of the *groEL* gene revealed a high degree of heterogeneity among and within *Anaplasma* sequences generated from the African buffalo [50]. Analysis of *A. marginale* 16S rRNA sequences identified four sequences that grouped into a distinct clade on phylogenetic analysis [50]. Additionally, a qPCR assay amplifying the *msp1β* gene detected *A. marginale* in 97.3% of cattle sampled from five districts in Mozambique, with sequence analysis revealing the presence of eight *msp4* and five *msp5* haplotypes of *A. marginale* circulating in the sampled animals [63]. Furthermore, use of the reverse line blot (RLB) hybridization assay, based on the 16S rRNA gene detected *A. marginale* in 20% of African buffalo screened from northern Botswana [8].

In North Africa, *A. marginale* was detected in 27.4% of cattle in Tunisia using a conventional duplex PCR assay targeting the *msp4* gene of *A. marginale* and the *msp2* gene of *A. phagocytophilum* [64]. Another molecular study found the annual prevalence of *A. marginale* infection to be 4.7% in sampled cattle [43]. Subsequent sequencing of an 805 bp fragment of the *msp4* gene revealed two distinct genotypes of *A. marginale* circulating in cattle in Tunisia that showed a high sequence homology with other *A. marginale* sequences from other African countries [43]. Use of a duplex qPCR assay targeting the *msp1*β gene detected *A. marginale* in 25.4% of cattle screened from three localities in the country [65]. Sequencing and analysis of the *msp4* gene identified the presence of nine *msp4* sequence variants of *A. marginale* [65]. The high genetic variation seen in *A. marginale msp4* sequences was attributed to the continuous introductions of infected animals from diverse sources into the study area [65]. Cattle breed, climatic conditions, husbandry practices and tick infestation were found to be risk factors that contributed significantly to *A. marginale* prevalence [65]. A phylogeographic characterization of *A. marginale* in blood samples collected from cattle across 11 governorates in Tunisia using the *lipA* and *sucB* genes identified five *lipA A. marginale* genotypes and a single *sucB* genotype circulating in the cattle [56]. Sequencing of the OmpA protein vaccine candidate also identified two *A. marginale* genotypes [56]. The study found that cattle from subhumid bioclimatic regions, female cattle and tick-infested cattle had statistically higher *A. marginale* prevalence [56]. Another study in the country characterized *A marginale* in cattle from seven districts with single-gene analysis and multilocus sequence typing (MLST) of the *dnaA*, *ftsZ*, *groEL*, *lipA*, *recA*, *secY* and *sucB* loci [57]. Sequence analysis identified seven *A. marginale* genotypes of the *dnaA*, *ftsZ* and *recA* genes, five genotypes of the *groEL* and *lipA* genes, three genotypes of the *secY* gene and four genotypes of the *sucB* gene [57]. The high genetic diversity of *A. marginale* strains in the study was similarly attributed to the practice of importing live cattle into the country from different regions and the distribution of infected ticks by wild ruminants and migrating birds [57].

In Egypt, *A. marginale* was first detected in *Hyalomma anatolicum* and *Rhipicephalus annulatus* using a qPCR assay based on the 16S rRNA gene, then subsequently characterized using the 16S rRNA and *msp5* genes [48]. *A. marginale* DNA was also detected using a 16S RNA gene PCR in two ticks collected from cattle in the country [49]. In another study, the overall prevalence of *A. marginale* was 21.3% in cattle, with detection rates of 14.1% in acutely ill cattle and 24.7% in apparently healthy animals using qPCR targeting the *msp1β* gene of *A. marginale* [46]. Positive samples were confirmed by 16S rRNA gene sequencing [46]. The higher detection rate of *A. marginale* in asymptomatic animals suggested these were carrier animals that act as reservoirs of infection for ticks to transmit the agent to susceptible animals [46]. Besides that, *A. marginale* was also detected in 15.2% of cattle and 1.2% of water buffaloes using *groEL* gene PCR where sequence analysis showed that *A. marginale groEL* sequences in the cattle displayed 98% similarity [55]. In addition, *A. marginale* sequences from buffaloes differed by 12 amino acid substitutions in comparison to the cattle sequences suggesting significant *A. marginale* strain diversity in the study area of Menoufia, Egypt [55].

In another study in Egypt, *A. marginale* was detected in 95% of cattle, 28.5% of *Hyalomma excavatum* and 18% of *R. annulatus* sampled from three cities in the country using an RLB hybridization assay, conventional 16S rRNA and *msp1α* gene PCRs and sequencing [42]. Further research in the country detected *A. marginale* in 68.3% of cattle and 29.4% of buffaloes using *msp1β* gene qPCR [66]. A lower *A. marginale* prevalence of 50.2% in cattle and 42.5% in buffaloes was found using the RLB assay underlining the importance of using appropriate diagnostic tests for epidemiological studies [66]. Positive samples were sequenced using the *msp1α* gene, with analysis of *msp1α* microsatellite sequences showing the presence of 15 *A. marginale* genotypes circulating in cattle and buffaloes in the study areas [66]. In Algeria, *A. marginale* was detected in 11.4% of cattle screened using a 23S rRNA gene qPCR [54]. Positive samples were confirmed using conventional 16S rRNA gene PCR and sequencing [54]. In Sudan, a molecular prevalence study detected *A. marginale* in 10.7% of cattle screened using a 16S rRNA gene PCR and *msp4* gene sequencing [67].

For the west and central African region, in Nigeria, use of *msp4* and *msp2* gene PCRs detected *A. marginale* in 23% and 15.6% of blood samples collected from 275 cattle [13]. Positive samples were confirmed by sequencing [13]. The study reported several haplotypes of *A. marginale* circulating in the animals with the occurrence of mixed haplotypes circulating in some individual animals [13]. Furthermore, in the north–central region of the country, *A. marginale* was detected in 39.1% of 704 indigenous cattle using an RLB hybridization assay based on the 16S rRNA gene [21]. *A. marginale* was previously detected from the same region in *Rhipicephalus decolaratus* picked off cattle using 16S rRNA gene PCR and sequencing [52].

In Côte d’Ivoire, 23S rRNA gene qPCR and standard PCR were used to screen 378 ticks for tick-borne pathogens, detecting *A. marginale* in 0.5% of *Rhipicephalus microplus* [23]. Tick vectors associated with the transmission of *A. marginale* in Côte d’Ivoire included *Hyalomma rufipes*, *R. microplus*, *R. decoloratus* and *R. annulatus* [47]. A molecular survey for tick-borne pathogens in cattle in Benin found 52.7% of animals positive for *A. marginale* using *msp5* gene PCR [41]. Positive samples were additionally sequenced using the *msp5*, *msp4*, and *groEL* genes [41]. Sequence analysis showed *groEL* gene sequences were conserved while several polymorphisms were seen in *msp4* and *msp5* gene sequences, indicating the presence of multiple strains of *A. marginale* circulating in the country [41]. In northern Cameroon, use of 16S rRNA gene PCR and sequencing detected *A. marginale* in 21.9% of sampled cattle [29].

In East Africa, a molecular survey of tick-borne agents in blood samples collected from cattle in Pemba Island, Tanzania detected *A. marginale* in 15.9% of cattle using *msp5* gene PCR and sequencing [68]. In Tanzania, *R. microplus* is incriminated as the major vector transmitting *A. marginale* in cattle in the coastal and lake regions [68]. Phylogenetic analyses revealed that the *msp5* gene was conserved among field isolates from the different geographic locales [68]. Similar results were observed when *A. marginale* was detected in 10.2% of cattle sampled in Zanzibar using *msp5* gene PCR and sequencing [69]. In Kenya, *A. marginale* infection in cattle is endemic. Molecular screening for tick-borne pathogens in cattle from two farms found the average prevalence of *A. marginale* to be 7.9% using *msp5* gene PCR and sequencing [40]. Sequence and phylogenetic analyses showed a similar pattern to what was observed in Tanzania [68], with *A. marginale msp5* gene sequences obtained from cattle showing a high degree of conservation [40]. A possible explanation for this similarity could be that the same primer set was used for both studies, with the primers amplifying a conserved region of the *msp5* gene.

*A. marginale* was detected in a mere 0.6% of zebu cattle in Lambwe Valley in Kenya using PCR high-resolution melting (PCR-HRM) and 16S rRNA gene sequencing [16]. The agent was likewise detected in 31% of apparently healthy dairy cattle from a peri-urban area in the country using primers that amplified a 425 bp fragment of the 16S rRNA gene, with positive samples confirmed by sequencing [53]. *A. marginale* sequences obtained in the study were highly conserved, with 97.6 to 100% nucleotide similarity [53]. Furthermore, *A. marginale* was detected in 4.9% of cattle from livestock markets and slaughterhouses in western Kenya using PCR-HRM and 16S rRNA gene sequencing [45]. In the study, exotic breeds of cattle were found to be more likely infected with *A. marginale*, suggesting an innate resistance to *A. marginale* infection in indigenous breeds [45]. The presence of ticks was also an important predictor of *Anaplasma* species [45]. The study found a higher prevalence of *A. marginale* infection in cattle from slaughterhouses compared to the livestock markets, suggesting that farmers were more likely to dispose of sick animals via slaughter rather than selling them at the livestock markets [45]. In Uganda, *A. marginale* was detected in 19.2% of cattle sampled from a wildlife–livestock interface in the western region of the country using species-specific *groEL* gene PCR and sequencing [44]. 

Current data suggest that the msp genes are reliable genetic markers for *A. marginale*, with sufficient variation to establish phylogeographic patterns. Multiple *A. marginale* genotypes have been identified in wild ruminants across South Africa, Mozambique, and Egypt, based on analysis of the 16S rRNA, *groEL*, *msp4*, *msp5*, and *msp1α* genes. These findings highlight the importance of wildlife as reservoir hosts for *A. marginale* infection. Notably, *groEL* sequences of *A. marginale* in southern Africa were more heterogeneous than those found in other regions of Africa. Similarly, in East Africa, *msp5* sequences were found to be more conserved than those from other parts of the continent. Tick vectors associated with the transmission of *A. marginale* in Africa belong mainly to the genera *Rhipicephalus* and *Hyalomma*. High tick infestation and cattle breeds are significant risk factors for *A. marginale* infection in Africa, with exotic breeds showing greater susceptibility to the infection. The combination of single-gene and multilocus sequence analysis provides a better understanding on the diversity and evolution of *A. marginale* strains.

### 2.2. Anaplasma centrale

*Anaplasma centrale* is less pathogenic than *A. marginale* and usually does not cause any clinical signs in infected animals. It was discovered by Arnold Theiler in 1911, where he described the organism as being centrally located in the erythrocytes of host animals [70]. It is used as a live vaccine against *A. marginale* in several countries [4]. Studies have linked *Rhipicephalus simus* and *Dermacentor andersoni* as being competent to transmit *A. centrale* [71,72]. Infection with *A. centrale* imparts long-lasting protective immunity against some virulent strains of *A. marginale* [73]. The genetic diversity of *A. centrale* strains in Africa has been studied using the *msp1aS*, *msp4*, *msp5*, *groEL*, 23S rRNA and 16S rRNA genes [21,23,29,43,50,67,74] (Table 1). In South Africa, a new genotyping approach for *A. centrale* based on the *msp1aS* protein, which is a homologue of *A. marginale msp1α*, identified 32 *A. centrale* genotypes for the first time circulating in cattle, wildebeest and buffalo in the country that were clearly distinct from the vaccine strain [74]. The study suggested that wildlife in South Africa are reservoirs for *A. centrale* infection [74]. A follow-up study by the same group used 16S rRNA, *groEL* and *msp4* gene PCR and sequencing to characterize *A. centrale* in DNA from blood samples collected from African buffalo, waterbuck, eland, black wildebeest, blue wildebeest and cattle [62]. The authors found four *A. centrale* 16S rRNA and *mps4* genotypes and a single *A. centrale groEL* genotype circulating in the sampled animals [62].

In Botswana, *A. centrale* was detected in 30% of African buffalo screened using 16S rRNA gene-based RLB hybridization assay [8]. Additionally, four sequences of *A. centrale* have been detected in African buffalo from Mozambique using 16S rRNA and *msp5* gene sequencing [50]. In north–central Nigeria, *A. centrale* was detected in 6.3% of cattle using an RLB hybridization assay that targeted 16S rRNA gene probes [21]. *A. centrale* was also detected in 7.8% of zebu and taurine cattle sampled from northern Cameroon using 16S rRNA gene PCR and sequencing [29]. In Côte d’Ivoire, *A. centrale* was detected in 0.2% of *Amblyomma variegatum* using 23S rRNA gene qPCR and conventional PCR [23]. In Sudan, *A. centrale* was detected in 2.04% of cattle tested using 16S rRNA gene PCR and sequencing of the *msp4* gene [67]. The study found a significantly higher prevalence of *Anaplasma* spp. infection in cattle in the summer, which could be attributed to the proliferation of the tick vectors during the hotter months [67]. In Tunisia, a longitudinal survey found an average infection rate of *A. centrale* to be 7% in sampled cattle [43]. Subsequent sequencing of a 383 bp fragment of the 16S rRNA gene revealed two 16S rRNA gene variants of *A. centrale* circulating in cattle that were similar to the *A. centrale* vaccine strain detected in other cattle from sub-Saharan Africa [43]. Other research in Tunisia detected *A. centrale* in 15.1% of cattle from three localities using a duplex qPCR assay that amplified the *groEL* gene [65]. Sequencing and analysis of a 551 bp region of the 16S rRNA gene identified six sequence variants of *A. centrale* circulating in the cattle [65]. Tick-infested cattle, cattle from subhumid regions and cattle reared under traditional husbandry practices were significantly more infected by *A. centrale* [65]. Holstein breeds were also found to be less infected by *A. centrale* [65]. This was suggested to be due to a genetic resistance of the breed to this disease agent [65]. In summary, studies detecting *A. centrale* in Africa suggest that wild ruminants serve as reservoirs for the infection. While *A. centrale* may circulate in wildlife through natural tick transmission cycles, the exact role of ticks in transmitting *A. centrale* in Africa is not fully understood, and more research is needed. The *msp1aS*, 16S rRNA, and *msp4* genes have proven to be useful genetic markers for characterizing *A. centrale* infections in both cattle and wild ruminants in northern and southern Africa. Additional studies are necessary to examine the genetic diversity of *A. centrale* strains in other regions of the continent, providing further clarity on the epidemiology of *A. centrale* infection.

### 2.3. Anaplasma phagocytophilum and A. phagocytophilum like-Strains

*Anaplasma phagocytophilum* causes tick-borne fever in domestic and wild animals, canine granulocytic anaplasmosis in dogs, equine granulocytic anaplasmosis in horses and human granulocytic anaplasmosis (HGA) in humans [75]. Ticks of the *Ixodes* genus are the main vectors of *A. phagocytophilum* transmission in Europe, the United States and Asia [76]. The reservoir hosts of *A. phagocytophilum* include the white-tailed deer, white-footed mouse, dusky-footed woodrats, squirrels, chipmunks and raccoons in the United States [77] and the roe deer, red deer, and yellow-necked and wood mice in Europe [78,79]. Even though morbidity and mortality of *A. phagocytophilum* are generally low in animals, economic losses due to reduced milk yield, decreased weight gain, abortion and infertility have been incurred by livestock farmers [75,76]. Fever, chills, headache and muscle aches are some of the clinical signs of HGA infection in humans [80,81,82,83]. Tetracycline has been used successfully in the treatment of HGA [76,84], while rifampin is used as a substitute drug for treatment in individuals that are allergic to tetracyclines [85,86]. Doxycycline hyclate is another drug that has been used successfully in the treatment of HGA [75]. The administration of long-acting antibiotics such as tetracycline as prevention before the transfer of animals from areas devoid of tick vectors to tick-infested grazing land has been recorded [84].

Genetic markers used in the characterization of *A. phagocytophilum* in Africa include the *msp2*, *msp4*, citrate synthase (*gltA*), *groEL*, 16S rRNA and 23S rRNA genes [11,14,22,50,64,87,88,89,90,91,92] (Table 1). In Tunisia, *A. phagocytophilum* was detected in 0.6% of cattle using a duplex PCR assay that amplified the *msp2* gene [64]. The organism was also detected in 13.6% of *Hyalomma aegyptium* ticks obtained from tortoises in the country using a nested PCR that amplified a 641 bp fragment of the 16S rRNA gene [89]. Sequence analysis identified two 16S rRNA gene variants of *A. phagocytophilum* in *Hy. aegyptium* that shared 99.7% sequence similarity and differed by two nucleotide substitutions [89]. Other research in the country detected *A. phagocytophilum* from the spleen of a wild rodent *Rattus rattus* using 16S rRNA gene PCR and sequencing [90]. In yet another study in Tunisia, use of nested 16S rRNA gene PCR detected an *A. phagocytophilum*-like sp. in 3.9% of sheep, 2.5% of goats and 0.5% of cattle sampled [93]. Restriction fragment length polymorphism (RFLP) further identified two unique strains of the organism [93]. Sequencing of a partial 16S gene fragment identified two sequence variants each of the *A. phagocytophilum*-like sp. from each strain of the organism present in sheep and goats in the country [93].

The use of 16S rRNA gene-based PCR-RFLP in combination with sequencing and phylogenetic analysis revealed *A. phagocytophilum*-like sp. in *Rhipicephalus turanicus* collected from goats and sheep in the country [10]. Other research using the same molecular technique detected *A. phagocytophilum*-like 1 and 2 strains in sheep and goats in the country [92]. Sequencing and analysis of the 16S rRNA and *groEL* genes identified two 16S and 20 *groEL* sequence types of *A. phagocytophilum*-like 1 and 2 strains circulating in the small ruminants [92]. The authors suggested that *Rhipicephalus* ticks may be the vectors responsible for the transmission of *A. phagocytophilum*-like 1 and 2 strains in the region [92]. Furthermore, a molecular survey of small ruminants in Tunisia reported the detection of an *Anaplasma* sp. genetically related to *A. phagocytophilum* using 16S rRNA gene PCR and sequencing in 47.5% of goats and 7.7% of sheep [94]. Sequence analysis revealed four 16S rRNA genotypes of this novel *A. phagocytophilum*-like sp. in goats and three genotypes in sheep [94]. In Algeria, use of a 23S rRNA gene qPCR and sequencing of the 23S rRNA and 16S rRNA genes identified *A. phagocytophilum* in 71.4% of sequences from cattle [11]. Subsequent sequence analysis revealed three sequence variants of *A. phagocytophilum* circulating in cattle based on the two genetic markers used [11].

In Ethiopia, a molecular survey that screened blood samples obtained from cattle using 16S rRNA gene PCR-RFLP with the enzymes, *Mbo*II, *Hha*I and *Msp*I detected *A. phagocytophilum* in 2.7% of the cattle samples [22]. In Zambia, an *Anaplasma* sp. sequence with 100% identity to *A. phagocytophilum* was detected in 13.6% of vervet monkeys and baboons using 16S rRNA gene PCR and sequencing [88]. Given that the sequence length was only 305 bp, sequence data from other genetic markers was needed for definitive species classification [32].

In South Africa, *A. phagocytophilum* near full length 16S rRNA gene sequences was obtained from three dogs and a rodent (*Mastomys natalensis*) in a rural community in Mpumalanga Province using 16S rRNA gene PacBio circular consensus sequencing [14]; 16S rRNA gene sequences with fragment lengths between (690–693 bp) were also obtained from two rodents (*M. natalensis* and *Rattus tanezumi*) and an acute febrile illness patient from the community [14]. Sequence analysis indicated the presence of two 16S rRNA gene sequence variants and one *gltA* gene sequence variant of *A. phagocytophilum* circulating in dogs and rodents in the study area [14]. *A. phagocytophilum* DNA was additionally detected from a pool of *Haemaphysalis elliptica* collected from urban stray dogs in the country using 16S rRNA gene PCR and sequencing [91].

In Zimbabwe, a 16S rRNA gene PCR and sequence analysis of samples from captive wild felids found *A. phagocytophilum* infection in 50% of servals, 13% of wild cats and 7% of lions [87]. The primers used in the study amplified a 478 bp fragment of the 16S rRNA gene therefore as previously mentioned, these sequences may not have sufficiently covered variable regions since minor nucleotide differences exist in the 16S rRNA gene between closely related *Anaplasma* species [14]. In Mozambique, a sequence of *A. phagocytophilum* was detected from 16S rRNA gene sequencing of samples from the African buffalo [50]. In Angola, two *A. phagocytophilum* sequences were detected in cattle using 16S rRNA gene PCR and sequencing in Huambo Province [24]. In summary, *A. phagocytophilum* was detected in a wide range of animals that included cattle, sheep, goats, dogs, wild rodents, baboons, wild felids, and buffalo. It is unclear whether these were competent *A. phagocytophilum* reservoir hosts or spillover hosts, as this information remains unknown. There is still limited information on the tick vectors associated with *A. phagocytophilum* transmission on the continent, as the agent has been detected in *Hy. aegyptium*, *R. turanicus* and *H. elliptica*, and thus more studies on tick vectors are needed. Although, the 16S rRNA gene has a limited ability to discriminate between *Anaplasma* species, it proved to be a useful genetic marker in the documented studies, as two *A. phagocytophilum* variants were identified in dogs and rodents in South Africa and in *Hy. aegyptium* in Tunisia. In addition, three 16S and 23S rRNA sequence variants were identified in cattle in Algeria. The *groEL* gene proved its usefulness as a suitable genetic marker differentiating between *A. phagocytophilum*-like 1 and 2 strains in small ruminants in Tunisia. Most of the studies that reported detection of *A. phagocytophilum* were in northern and southern Africa; therefore, more studies in other geographical regions in wildlife and ticks using single-locus genes such as the *ank*, *groEL*, *gltA* and *drhm* are recommended. The use of multilocus sequence analyses and whole-genome sequencing is also required to uncover the epidemiological cycle and phylogeny of this important zoonotic agent.

### 2.4. Anaplasma platys and A. platys-like Strains

*Anaplasma platys* is the cause of canine infectious cyclic thrombocytopenia [5]. It is the sole rickettsial species that is known to cause infection in host platelets [5]. The dog is regarded as the natural host for *A. platys* [95] while *R. sanguineus* sensu lato (s.l) is presumed to be the vector responsible for its transmission in Africa [96]. *Anaplasma platys* infection can present as a subclinical infection with negligible clinical signs; however, in some cases, clinical signs have been reported in dogs [97,98]. *Anaplasma platys* was suggested as a zoonotic agent based on two studies that documented clinical infection in humans [99,100].

Genetic markers used in the detection and characterization of *A. platys* and *A. platys*-like strains in Africa include the 16S rRNA, 23S rRNA, *groEL* and *gltA* genes [11,13,14,15,16,18,21,29,42,44,45,48,50,53,55,63,101,102,103,104,105,106,107,108,109,110] (Table 1). The first report of *A. platys* detection in Africa was in the Democratic Republic of the Congo (DRC), where the agent was detected in an apparently healthy dog and in *Rhipicephalus sanguineus* using 16S rRNA gene PCR [96]. Subsequent sequencing of positive samples was done using the *groEL* and *gltA* genes [96]. Likewise, the organism was detected in 36.6% of cattle sampled from Cameroon using 16S rRNA gene PCR and sequencing [29]. In the study, age was found to be a risk factor for *A. platys* infection as older animals were more likely to be infected [29]. In Nigeria, *A. platys* 16S rRNA gene species-specific primers detected the organism in 20% of cattle screened. Ensuing use of the *groEL* gene detected the organism in 45.9% of the animals [13]. The study reported several haplotypes of *A. platys* circulating in the cattle [13]. *Anaplasma* platys was also detected in 61% of camels in northwestern Nigeria using the RLB hybridization assay and sequencing of the 16S rRNA gene [106]. The authors also detected the agent in 3.9% of cattle from the north–central region of the country [21]. Additionally, an *A. platys*-like organism was detected in 6.6% of dogs and 1.9% of *R. sanguineus* collected from the dogs across four states in the country using 16S rRNA gene qPCR and sequencing [104]. In Cape Verde, *A. platys* was detected in 34.6% of indigenous apparently healthy dogs using 16S rRNA gene primers specific for members of the Anaplasmataceae family and *A. platys* [105]. The results were, however, not confirmed by sequencing [105]. In Côte d’Ivoire, *A. platys* was detected in 8.5% of dogs, 37.7% of *R. sanguineus*, 16.9% of *Haemaphysalis leachi* and 0.8% of *Hyalomma* and *Amblyomma* spp. using 16S rRNA gene PCRs and sequencing [107]. In Senegal, *A. platys* was detected in 15.6% of dogs using 23S rRNA gene qPCR and sequencing of the beta subunit of the RNA polymerase (*rpoB*) gene [28].

In Egypt, *A. platys*-like sequences were obtained from *R. annulatus* using 16S rRNA gene PCR and sequencing [48]. In another study, *A. platys* had a minimum infection rate (MIR) of 0.25% and 1.2% in *Hy. excavatum* and *R. annulatus*, respectively, using RLB hybridization, 16S rRNA gene PCRs and sequencing [42]. Use of 16S rRNA gene sequencing in additional research detected *A*. *platys*-like sequences in 14.1% of cattle [55]. Likewise, other research in the country detected *A. platys* in cattle and buffaloes from three regions using 16S rRNA and *groEL* gene sequencing [66]. *A. platys* has also been detected in cattle from Algeria using 23S rRNA real-time PCR and confirmed by 23S rRNA and 16S rRNA gene sequencing [11]. Furthermore, the organism was detected in 24% of *R. sanguineus* ticks picked off infested dogs in central and eastern Algeria using 16S rRNA gene qPCR [15]. *A. platys* was also detected in 7.5% of dogs sampled from four cities in Morocco using a commercial strain-specific qPCR assay [102]. An *A. platys*-like agent has been detected in 17.7% of Tunisian one humped camels using full-length 16S rRNA gene primers [103]. Analysis of the 16S rRNA gene sequences showed the presence of four sequence variants of the *Anaplasma* sp. circulating in the camels [103]. Use of a *groEL* gene-based PCR-RFLP assay detected *A. platys*-like strains in 5.6% of apparently healthy camels and 0.3% of *Hyalomma dromedarii* sampled from five governorates in the country [111]. Sequencing and analysis of the 16S rRNA and *groEL* genes identified three 16S rRNA and six *groEL A. platys*-like genotypes circulating in the camels [111]. A single 16S rRNA genotype was identified in *Hy. dromedarii* [111]. Camels from the arid and subarid regions were found to be significantly more infected with the *A. platys*-like strains than those sampled from the Sahara area. The authors suggested that this was because of the common practice of keeping camels together with other ruminants in the same shelter in arid and semiarid regions [111]. Since the platelets of the camels in the aforementioned studies were not infected [103,111], it has been recommended that further research through in vitro culture and experimental studies are required to understand the paradox of *A. platys*-like infection in camels [112]. In north Tunisia, an *A. platys*-like organism was detected in 3.5% of cattle, 11% sheep and 22.8% of goats using heminested *groEL* PCR, RFLP assay and sequencing [113]. The disparity seen in the infection rates in ruminants was suggested to be due to existing differences in host vulnerability and infestation rates by tick vectors [113]. The study identified nine *A. platys*-like *groEL* genotypes in sheep and goats [113]. Recently, *A. platys*-like strains were detected in 16.4% of goats and 15.3% of sheep in Tunisia using heminested *gltA* and *groEL* gene PCRs and sequencing [110]. The authors identified 22 unique sequence types of *A. platys*-like *gltA* gene sequences, indicating the high variability of the *gltA* gene [110].

In Kenya, *A. platys* was detected in 18.6% of dogs, 73.3% of *Rhipicephalus camicasi*, 1.2% of *R. sanguineus*, *R. simus* and *H. leachi*, 31.4% of *Rhipicephalus pulchellus*, 3.5% of *Rhipicephalus humeralis* and 3.5% of *Amblyomma* spp. sampled from the dogs using 16S rRNA gene PCR and sequencing [107]. Similarly, the agent was detected in 44.8% of dairy cattle in the country using 16S rRNA gene PCR and sequencing [53]. Obtained *A. platys* sequences in the study displayed multiple-nucleotide polymorphisms with the identification of six sequence variants of *A. platys* circulating in the cattle [53]. *A. platys* was then detected in *Rhipicephalus evertsi evertsi*, *Rhipicephalus pravus* and *R. pulchellus* sampled from domestic dogs in Baringo and Homa Bay counties in the country using 16S rRNA gene PCR-HRM analyses and sequencing [109]. In the study, *A. platys* was also detected in 19.5% of goats and 100% of dogs in Baringo county and in 12.9% of cattle, 6.6% of goats, 14.3% of sheep and 57.1% of dogs sampled from Homa Bay county [109]. *A. platys*-like sequences have been detected in 16.9% of zebu cattle in Kenya using PCR-HRM analysis and 16S rRNA gene sequencing [16]. Additionally, *A. platys*-like sequences were detected in 13.5% of cattle from livestock markets and abattoirs in western Kenya using PCR-HRM and 16S rRNA gene sequencing [45]. *A. platys*-like sequences have also been detected in *R. decolaratus* from cattle and *Am. variegatum* collected from a white rhinoceros in the country using 16S rRNA gene PCR and sequencing [108].

In South Africa, *A. platys* has been detected in *R. evertsi evertsi* using 16S rRNA gene-based RLB hybridization and sequencing [101]. In addition, nine 16S rRNA gene sequences of *A. platys* were obtained from two domestic dogs in Mpumalanga Province in the country [14]. Sequence analysis indicated *A. platys* sequences were conserved and identical to each other [14]. In Zambia, three *A. platys* sequences each of the 16S rRNA and *gltA* genes were detected from samples collected from peri-urban and rural domestic dogs [18]. In Mozambique, seven sequences of *A. platys* were also obtained using 16S rRNA gene sequencing in samples from African buffalo [50]. Further sequencing of the 16S rRNA and *groEL* genes in DNA from cattle blood samples from five districts in the country that had previously tested positive for *A. phagocytophilum* on *msp2* gene PCR indicated the presence of *A. platys*-like sequences in the cattle [63]. The possibility of the *msp2* gene qPCR assay for A. *phagocytophilum* cross-reacting with *A. platys* has been reported [14]. In Angola, three *A. platys* sequences were detected in cattle using 16S rRNA gene PCR and sequencing [24]. The vector and host range for *A. platys* in Africa may be wider than previously thought, as the organism was detected in cattle, goats, camels, buffaloes and multiple species of *Rhipicephalus*, *Haemaphysalis*, *Hyalomma* and *Amblyomma* ticks. More studies are clearly needed to clarify this point. Overall, the *groEL*, *gltA* and 16S rRNA genes were suitable genetic markers for the characterization of *A. platys* in Africa by identifying multiple sequence variants in Nigeria, Tunisia and Kenya. This was not the case in southern Africa, where *A. platys* sequences were mostly conserved. Previous in silico analyses of the *groEL* operon had suggested the use of two partial regions of the gene that were useful in delineating intraspecific diversity within the *Anaplasma* species [114]. For epidemiological studies, RFLP assay is a useful molecular tool for the detection and differentiation of coinfections of *A. platys* and *A. platys*-like agents in ticks, ruminants and cats that share similar hosts for these related bacteria [113].

### 2.5. Anaplasma ovis

*Anaplasma ovis* is a tick-borne bacterium of sheep, goats and wild ruminants and the cause of ovine anaplasmosis [115]. The disease has a worldwide distribution [116]. *A. ovis* usually causes a subclinical infection, but when subjected to stressful conditions, animals can develop the clinical disease, where signs such as fever, inappetence, lethargy, abortion and a reduction in milk production are seen [116]. *A. ovis* infection makes animals prone to other disease agents that can lead to a worsening condition and possibly death [116]. In Africa, *A. ovis* has frequently been detected in ticks of the *Rhipicephalus* genus [10,17,22,26,54,117] and less frequently in *Amblyomma* ticks [26,109]. Factors that impact the prevalence of *A. ovis* in small ruminants are suggested to include the sampling technique used, presence of tick vectors, livestock management practices, the climate and ecology of the study area and the immune status and vulnerability of the host animals [118]. Genetic markers used in the detection and characterization of *A. ovis* in animals and ticks in Africa include the 16S rRNA, 23S rRNA, *msp4*, *gltA*, *msp1a* and *groEL* genes, with a majority of the studies using the *msp4* gene [9,10,17,54,67,90,94,109,119,120,121,122,123] (Table 1).

In a longitudinal molecular survey in Tunisia, the average prevalence of *A. ovis* was 35.6% in sheep and 46% in goats [9]. Sequence analysis of *A. ovis msp4* gene sequences revealed one *A. ovis* genotype each in sheep and goats [9]. *Anaplasma ovis* was also detected in 93.8% of sheep and 65.3% of goats in the country using loop-mediated isothermal amplification (LAMP) that used six primers to amplify the *msp4* gene [94]. Sequencing of a 719 bp fragment of the *msp4* gene revealed five genotypes of *A. ovis* circulating in sheep and a single genotype in goats [94]. Sequencing and analysis of the *msp4* gene also identified a single *A. ovis* genotype in goats and five genotypes in sheep [94]. Additional research in Tunisia detected *A. ovis* in the spleen of *R. rattus* using 16S rRNA gene PCR, and positive samples were confirmed by *msp4* gene sequencing [90]. Phylogenetic analysis showed *A. ovis msp4* gene sequences grouping into two separate clusters [90]. Besides that, *A. ovis* was detected in 7.9% of *R. turanicus* and 2.5% of *R. sanguineus* collected from sheep and goats in the country using 16S rRNA gene PCR [10]. Subsequent multi locus genotyping of *A. ovis* with the 16S rRNA, *msp4* and *groEL* genes revealed the presence of two *A. ovis* 16S rRNA and *msp4* genotypes in *R. turanicus* and *R. sanguineus*. Furthermore, eight unique *groEL* genotypes six in *R. turanicus* and two in *R. sanguineus* were identified, five of which were reported as novel genotypes [10]. Recently in central Tunisia, the infection dynamics of *A. ovis* in sheep over a five-month period showed the molecular prevalence of *A. ovis* to be 22.6% in lambs and 100% in ewes at the first sampling and 26.3% in lambs and 85.7% in ewes at the second sampling using *msp4* gene PCR and sequencing [124]. The high prevalence in the ewes supported the existence of endemic stability of *A. ovis* in sheep in the region [124]. The authors speculated that the decrease in the *A. ovis* prevalence dynamics in ewes from 100% to 85.7% could be attributed to lower *A. ovis* burdens that occur outside the transmission system [124]. *A. ovis* was detected in 1.2% of camels sampled from seven camel herds across five localities in the country using *msp4* gene PCR [125]. Sequencing and analysis of the *msp4* and *groEL* genes identified two *msp4* and five *groEL A. ovis* genotypes in the camels [125]. The study suggested that the low infection rate *of A. ovis* in camels could be a result of accidental infection caused by close and prolonged contact with small ruminants such as sheep and goats that have significantly higher rates of *A. ovis* prevalence in the region [125]. In other research in Tunisia, genetic characterization of *A. ovis* isolates in goats, sheep, camel and *R. turanicus* by PCR and sequencing of the *gltA*, *groEL* and *msp1a* genes identified the presence of six *gltA*, 17 *groEL* and 18 *msp1a A. ovis* genotypes from the isolates [123]. The study found comparative typing of *A. ovis* to be better with the *groEL* gene when compared to the *gltA*, 16S rRNA and *msp4* genes [123]. Phylogenetic analysis found the N-terminal region of the Msp1a protein to be a very informative region for phylogeographic delineation thus the authors recommended the use of this gene for phylogeographic differentiation of *A. ovis* strains worldwide [123].

In Algeria, *A. ovis* was detected in *R. sanguineus* and *Rhipicephalus bursa* collected from sheep and goats and in the goats, sheep and cattle using 23S rRNA gene PCR and sequencing [54]. *A. ovis* was also detected in 52% of *R. bursa* and 22.7% of *R. turanicus* collected from sheep and in 61.7% of sheep and 54.2% of goats sampled in the northeastern region of the country using 23S rRNA gene qPCR, standard PCR and sequencing [117]. In Egypt, use of *msp4* gene PCR detected *A. ovis* in 9.1% of sheep screened [55]. Analysis of partial *A. ovis msp4* gene sequences showed sequences had a similarity index of 98.9–100% [55]. In Sudan, *A. ovis* was detected in 35.86% of goats, 32.5% of sheep and 0.5% of cattle screened using a PCR assay that amplified the 16S rRNA gene and positive samples were confirmed with *msp4* gene sequencing [67]. In Senegal, *A. ovis* was detected in 55.9% of sampled sheep using 23S rRNA gene qPCR and sequencing of the 23S rRNA, *rpoB* and 16S rRNA genes [28].

In East Africa, *A. ovis* has been detected in *R. decoloratus* and *R. evertsi evertsi* collected from cattle and sheep in Oromia, Ethiopia using 16S rRNA gene PCR and sequencing [120]. A survey of questing ticks at the Masai Mara National Reserve in Kenya detected *A. ovis* in *R. evertsi evertsi* and *Rhipicephalus appendiculatus* with an MIR of 200 and 0.89 using 16S rRNA PCR-HRM analysis and sequencing [17]. *A. ovis* was also detected in 34.2% of sheep samples from two counties in Kenya using *msp4* gene PCR with phylogenetic analysis showing the presence of multiple *msp4* genotypes of *A. ovis* circulating in the sheep [119]. Furthermore, *A. ovis* was detected in 88.3% of sheep, 14.3% of *Amblyomma gemma*, 8.3% of *Amblyomma lepidum*, 15.6% of *R. camicasi* and 100% of *R. pulchellus* collected from sheep in 12 sites in northern Kenya using 16S rRNA gene PCR-HRM and sequencing [26]. The same technique detected *A. ovis* in *Am. variegatum*, *Am. gemma*, *R. pulchellus* and *R. appendiculatus* parasitizing cattle, goats and sheep in Baringo and Homa Bay counties of the country [109]. In Baringo, *A. ovis* was detected in 15.6% of cattle, 5.7% of goats and 30.3% of sheep, while in Homa Bay it was detected in 3.2% of cattle, 3.3% of goats and 4.8% of sheep [109]. In Uganda, *A. ovis* was detected in 26.1% of sheep and 25.4% of goats sampled from a human–wildlife–livestock interface using 16S rRNA and *msp4* gene PCRs [126].

The use of *msp4* gene PCR detected *A. ovis* in 45.9% of goats and 16.7% of sheep sampled across two provinces in South Africa [121]. The study speculated that goats were more vulnerable to *A. ovis* infection than sheep [121]. Other research detected *A. ovis* in *Am. hebraeum* collected from donkeys using 16S rRNA gene PCR and sequencing [127]. In Botswana, a high *A. ovis* prevalence of 76% was reported in goats sampled from three different villages using *msp4* gene PCR and sequencing [122]. In conclusion, more *A. ovis* genotypes were identified using the *msp4*, *msp1a* and *groEL* genes compared to the 16S rRNA gene, indicating the usefulness of these genetic markers. Epidemiological surveys for the detection of *A. ovis* are recommended for the West African subregion, as there are currently very limited data available on its occurrence and prevalence.

### 2.6. Anaplasma bovis

*Anaplasma bovis* infects circulating monocytes and macrophages in the blood of host animals, usually domestic and wild ruminants [128]. In cattle, *A. bovis* infection is generally asymptomatic, except in some instances, where fever, anemia, debility, anorexia, enlarged lymph nodes, depression and occasional death have been reported [128,129]. The 16S rRNA gene is the only genetic marker used in the detection and characterization of *A. bovis* in ruminants and ticks in Africa [9,16,17,43,53,109,130] (Table 1).

In North Africa, a molecular survey of *A. bovis* in small ruminants in northern Tunisia showed the average prevalence for *A. bovis* to be 7.4% in sheep and 10.1% in goats [9]. Sequencing of the 16S rRNA gene from randomly selected sheep and goats revealed one genotype of *A. bovis* circulating in both sheep and goats, one genotype in sheep and another genotype in the goats [9]. *Anaplasma bovis* was also detected in 8.3% of *Hy. dromedarii* ticks collected from three scimitar-horned oryx from a nature reserve in the country using nested primers that amplified a 551 bp fragment of the 16S rRNA gene [130]. Furthermore, the average infection rate of *A. bovis* was found to be 4.9% in cattle sampled from five different governorates in the country [43]. Sequencing of the 16S rRNA gene indicated the presence of three distinct *A. bovis* sequence variants circulating in the cattle [43]. Other research in northern Tunisia detected *A. bovis* in 42.7% of sheep and 23.8% of goats from five localities and two bioclimatic areas using primary and nested PCRs of the 16S rRNA gene [131]. Sequencing and analysis of the 16S rRNA gene identified a single *A. bovis* genotype in goats and two genotypes in sheep [131]. Goats from the subhumid area had significantly higher prevalence of *A. bovis* infection [131]. This was suggested to be a possible consequence of bioclimatic conditions playing a role in the proliferation of tick vectors [131]. Additionally, *A. bovis* was detected in 3.9% of cattle screened from three localities in the country using nested 16S rRNA gene PCR and sequencing [65]. Sequence analysis identified three unique sequence variants of *A. bovis* circulating in the cattle [65]. The study found that cattle from subhumid areas, cattle reared under traditional management systems and cattle infested by ticks had significantly higher infection rates of *A. bovis* [65].

In Kenya, *A. bovis* was detected in 17.4% of cattle using PCR-HRM and confirmed by 16S rRNA gene sequencing [16]. *A. bovis* was also detected in 13.8% of apparently healthy dairy cattle using 16S rRNA gene PCR and sequencing [53]. The obtained *A. bovis* sequences had multiple-nucleotide polymorphisms with three identified sequence variants [53]. *A. bovis* was also detected in questing *R. appendiculatus* ticks from the Masai Mara nature reserve with an MIR of 0.89 using 16S rRNA gene PCR-HRM and sequencing [17]. The same technique detected *A. bovis* in *Am. gemma*, *Am. variegatum*, *R. evertsi evertsi*, *Hyalomma truncatum*, *Hy. rufipes*, and *Rhipicephalus praetextatus* sampled from livestock and in 17.8% of cattle, 6.8% of goats and 9.1% of sheep sampled in the country [109]. In Tanzania, *A. bovis* was detected in questing *R. praetextatus* collected from the Ngorongoro Crater using 16S rRNA gene PCR and sequencing [132].

In South Africa, *A. bovis* was detected in *Rhipicephalus* sp. near *warburtoni* collected from eastern rock sengi (*Elephantulus myurus*) in Limpopo province using 16S rRNA gene PCR and sequencing [133]. A follow-up study detected *A. bovis* in 28.6% of sengis using the same genetic marker with phylogenetic analysis of the 16S rRNA gene confirming the monophyly of *A. bovis* variants [134]. The authors found a massive infestation of *R.* sp. near *warbutoni* on *E. myurus* and concluded that *R.* sp. near *warbutoni* could be the vector of *A. bovis* in *E. myurus* [134]. The study further demonstrated that *E. myurus* is a natural reservoir for *A. bovis* in that geographic region [134]. Research in the same province also detected *A. bovis* in *R. evertsi evertsi* collected from donkeys using 16S rRNA gene PCR and sequencing [127]. Finally, *A. bovis* was detected from a cat in Luanda, Angola using 16S rRNA gene PCRs and sequencing, the first description of its occurrence in domestic cats outside of Japan [135]. There is still limited information on the epidemiology of *A. bovis* on the African continent. Molecular studies using genetic loci other than the 16S rRNA gene are recommended to determine the reservoir hosts and tick vectors of *A. bovis* so adequate control measures can be instituted.

### 2.7. Other Anaplasma *spp.* Detected in Africa

*Anaplasma capra* was first identified as a putative species using 16S rRNA and *msp4* gene sequences obtained from goats in central and southern China [136]. It was subsequently detected in hospital patients in Heilongjiang Province, China, that presented with flu-like symptoms in addition with regional lymphadenopathy, fever, vomiting, diarrhea and malaise [7]. *A. capra* was then provisionally named a novel tick-borne zoonotic *Anaplasma* sp. [7]. Since then, *A. capra* infection has been detected in three continents, with recorded infections in humans, ruminants, dogs, wild animals and a variety of ticks [137,138,139,140,141]. In Africa, there is only one published report of *A. capra* detection in which six sequences of *A. capra* were obtained from cattle sampled in Huambo, Angola using targeted 16S rRNA gene PCR and sequencing [24] (Table 1).

*Anaplasma* sp. SA dog was initially detected from three dogs in South Africa using 16S rRNA and *gltA* gene PCR and sequencing [19]. The agent was subsequently detected in domestic dogs sampled from a rural community in a human–wildlife interface in the country using an RLB hybridization assay of the 16S rRNA gene and targeted sequencing of the genetic marker [20]. A closely related agent named *Anaplasma* sp. ZAM dog was subsequently detected in apparently healthy dogs in Zambia using 16S rRNA and *gltA* gene PCRs and sequencing [18]. In South Africa, *Anaplasma* sp. SA dog was again detected in domestic dogs and *R. sanguineus* ticks using 16S rRNA and *gltA* gene PCR and sequencing [14] (Table 1). Sequence analysis identified the presence of two 16S rRNA gene sequence variants of the agent in dogs and *R. sanguineus* ticks in the study [14]. A *gltA* gene sequence variant of *Anaplasma* sp. SA dog was also described from a dog [14]. The organism was found to cross-react with a qPCR assay that was targeted to amplify the *msp2* gene of *A. phagocytophilum* [14]. Phylogenetic analysis performed on 16S rRNA and *gltA* gene sequences persistently clustered *Anaplasma* sp. SA dog and *Anaplasma* sp. ZAM dog into a definite clade that provided adequate delineation from other *Anaplasma* species to justify classification as a different species [14]. The authors suggested that the novel organism be referred to as *Anaplasma* sp. SA dog and speculated that *R. sanguineus* could be the tick vector responsible for its transmission in southern Africa [14].

The same study also reported the detection of 16S rRNA gene sequences of *Candidatus* Anaplasma boleense in a heifer and *Anaplasma* sp. Mymensingh sequences from two cattle samples, the first description of both organisms in South Africa [14]. *Candidatus* Anaplasma boleense has subsequently been detected in cattle and sheep in Senegal using *groEL* gene sequencing [142] (Table 1). An *Anaplasma* sp. was detected in 7% of *R. evertsi evertsi*, *R. decoloratus*, *Amblyomma hebraeum* and *Rhipicephalus* spp. ticks collected from cattle, sheep and goats across four provinces in South Africa using 16S rRNA gene PCR and sequencing [143]. An ensuing study by the same group detected an *Anaplasma* sp. in *Am. hebraeum*, *H. elliptica* and *R. sanguineus* picked off dogs and cats in three provinces in the country using the 16S rRNA gene primers that was previously used [144] (Table 1).

Molecular characterization of 16S rRNA and *groEL* sequences revealed the presence of a novel organism *Candidatus* Anaplasma sphenisci associated with cytoplasmic inclusions in the erythrocytes of blood smears from the African penguin (*Spheniscus demersus*) in South Africa [31] (Table 1). Phylogenetic analysis showed that the organism belonged to the genus *Anaplasma* and was most closely related to the cluster that encompasses *A. marginale*, *A. centrale*, *A. ovis* and *A. capra* [31]. *Anaplasma sp.* was also detected in 100% of *R. microplus*, 92% of *R. evertsi evertsi*, 50% of *Hy. rufipes* and *Otobius megnini* and 40% of *R. decolaratus* sampled from cattle, donkey, horses, goats, sheep and vegetation from 10 districts in Lesotho using 16S rRNA gene PCR and sequencing [145]. Two putative *Anaplasma* spp. were additionally detected in 63% of *Argas walkerae* and 82.2% of *Ornithodoros moubata* collected from a chicken coop and African warthog burrows in a national park in Zambia using 16S rRNA gene PCR and sequencing of the 16S rRNA and *groEL* genes [146] (Table 1). Sequence analysis showed that obtained 16S rRNA and *groEL* gene sequences from *Ar. walkerae* were identical [146]. In the same vein, identical 16S rRNA gene sequences were obtained from *O. moubata* [146]. Partial *Anaplasma groEL* gene sequences from *O. moubata* indicated the presence of two sequence variants that differed by 10 nucleotide bases [146]. Phylogenetic analyses of 16S rRNA and *groEL* gene sequences showed that the novel *Anaplasma* spp. from *O. moubata* was closely related to *Ca.* Anaplasma sphenisci detected in the African penguin in South Africa [146].

*Anaplasma* sp. Omatjenne was first detected in blood samples from healthy Boer goats in the Northern Cape Province of South Africa [6]. It was subsequently detected in 6.47% of blood samples from cattle across five countries—Ethiopia, Côte d’Ivoire, Zambia, Rwanda and Morocco—using 16S rRNA PCR and RFLP [22]. In Nigeria, the agent was detected in 34.7% of cattle from the north–central region using an RLB hybridization assay [21] (Table 1). *Candidatus* Anaplasma camelii was detected in 40.3% of blood samples collected from one-humped camels across three states in northwestern Nigeria using semi-nested 16S rRNA gene PCR and sequencing [27]. Sequence analysis identified one haplotype of *Ca.* A. camelii circulating in the camels that differed from *A. platys* by a single deletion [27]. *Candidatus* Anaplasma camelii was also detected in 78.72% of apparently healthy camels, 2.72% of *Hy. dromedarii*, 3.33% of *Hy. rufipes*, 2.72% of *Hyalomma impeltatum*, 4% of *Hy. truncatum*, 8.5% of *Am. gemma*, 6% of *Am. lepidum*, 8.33% of *R. camicasi* and 6.7% of *R. pulchellus* collected from camels across 12 sites in northern Kenya using 16S rRNA gene PCR-HRM analysis and sequencing [26]. The organism was later detected in 2.2% of *R. camicasi* collected from co-grazing sheep in the study [26] (Table 1). Additionally, in West Africa, a novel *Candidatus* Anaplasma ivorensis was detected in two *Am. variegatum* ticks and a *R. microplus* tick in Côte d’Ivoire. Sequences were obtained from the 23S rRNA gene of Anaplasmataceae [23] (Table 1). *Candidatus* Anaplasma turritanum and *Ca.* Anaplasma cinensis were detected in domestic ruminants in Senegal using nested *groEL* and *gltA* gene PCRs and sequencing [142]. *Ca.* Anaplasma turritanum was detected in 62% of sheep and 32% of goats while *Ca.* Anaplasma cinensis was only detected in cattle [142]. A single-sequence variant of *Ca.* Anaplasma turritanum based on the *groEL* and *gltA* genes was found circulating in sheep and goats in the study [142] (Table 1). In Tunisia, phylogeny of *groEL* and *gltA* gene sequences obtained from goats and sheep recommended the reclassification of *Ca.* Anaplasma turritanum for all *A. platys*-like strains originating from the Mediterranean region [110]. A separate study in Senegal detected *Candidatus* Anaplasma africae in 3.7% of sheep, 10.3% of goats and 8.1% of cattle using a 23S rRNA qPCR and sequencing of the 23S, 16S rRNA and *rpoB* genes [28]. Furthermore, an *Anaplasma* sp. G75 was detected in two *Ixodes aulacodi* ticks picked from the greater cane rat *Thryonomys swinderianus* in Ghana using primary 16S rRNA gene PCR and nested PCRs targeting the *gltA* and *groEL* genes of Anaplasmataceae [25] (Table 1). The *gltA* and *groEL Anaplasma* sequences had 78.8% and 89.7% similarity to the sequence of *A. phagocytophilum* detected in a dog in Japan [25].

In Kenya, an uncharacterized *Anaplasma* sp. was detected in 40.8% of sampled sheep using 16S rRNA gene PCR and sequencing [119] (Table 1). The primers amplified partial fragments (335–430 bp) of the 16S rRNA gene [119]. A molecular survey of ticks collected from domestic and wild animals and vegetation detected an *Anaplasma* sp. in *R. pravus* from sheep in Kenya and in *R. decolaratus* collected from cattle in Ethiopia using partial primers that amplified 925 bp of the 16S rRNA gene [108] (Table 1). Positive results were confirmed by sequencing [108]. An *Anaplasma* sp. Lambwe was detected in 11.6% of zebu cattle in the country using PCR-HRM and sequencing of the 16S rRNA gene [16]. The *Anaplasma* sequence was identical with other presumed novel species—*Anaplasma* sp. Saso, *Anaplasma* sp. Dedessa and *Anaplasma* sp. Hadesa—detected in cattle in Ethiopia using PCR-RLB and sequencing of the 16S rRNA gene [30] (Table 1). Furthermore, three unidentified *Anaplasma* sp. sequences were detected from dairy cattle in Kenya using 16S rRNA gene PCR and sequencing [53]. *Anaplasma* sp. Hadesa was also detected in 7.8% of cattle in Cameroon using 16S rRNA gene PCR and sequencing [29] (Table 1).

An unclassified *Anaplasma* sp. was detected in 0.5% of *Amblyomma cohaerens* sampled from cattle in Adama, Ethiopia using 16S rRNA gene PCR [147]. Another unclassified *Anaplasma* sp. was detected in 32% of spotted hyenas sampled from Tanzania and in 100% of spotted hyenas and 82.4% of brown hyenas from Namibia using PCR primers that amplified a partial fragment of the 16S rRNA gene [148]. Use of 16S rRNA gene PCR also detected an *Anaplasma* sp. in 4% of *Am. gemma* collected from slaughter cattle and buffalo in the Iringa region of Tanzania [149] (Table 1).

In Algeria, an *Anaplasma* sp. was initially detected in blood samples from cattle using a 23S rRNA gene qPCR, and sequencing of the 23S rRNA and 16S rRNA genes [11]. In Tunisia, use of 16S rRNA gene PCR detected an *Anaplasma* sp. in 50% of *Hy. dromedarii* collected from scimitar-horned oryx in the Oued Dekouk nature reserve [130] (Table 1).

In Gabon, a molecular survey in organs of captured rodents using a 23S rRNA gene qPCR detected an *Anaplasma* sp. from 1.8% of *Ra. rattus* from central district, 14.8% of *Lemniscomys striatus*, 5.88% of *Praomys* sp., 3.7% of *Ra. rattus* and 5.3% of shrews captured from the peripheral district and in 14.8% of *L. striatus*, 3.7% of *Lophuromys* sp. and 11.8% of *Praomys* sp. trapped from vegetation areas [150] (Table 1). Positive samples were confirmed using nested PCR and sequencing of a longer region of the 23S rRNA gene [150]. The 23S rRNA sequences obtained in the study had 91–92% similarity with *A. phagocytophilum* previously detected from bovines in Algeria [11]. In summary, the 16S rRNA gene was the most utilized genetic marker used in the identification of these novel *Anaplasma* spp. Future studies using other genetic loci and whole-genome sequencing are recommended to unveil the diversity of *Anaplasmataceae* in Africa. This information would help to uncover the zoonotic potential of these putative species and determine their impact on veterinary and human health.

**Table 1:** Molecular epidemiology of various *Anaplasma* spp. detected in animal hosts and tick species in African countries.

*Anaplasma* sp.	Molecular * Method	Target Gene	Amplicon Length (bp)	Sequencing	Host or Vector	Country	Reference
*A. marginale*	PCR	*msp1α*	630–1200	Yes	Cattle	South Africa	[61]
	PCR	*msp1α*	630–1200	Yes	Cattle	South Africa	[59]
	qPCR	*msp1β*	419	Yes	Cattle	South Africa	[60]
	PCR	*groEL*	522	Yes	Cattle	South Africa	[60]
	PCR	*msp5*	195	Yes	*R. decoloratus*	South Africa	[12]
	qPCR	*msp1β*	95	No	Cattle	South Africa	[51]
	PCR	*msp1α*	630–1200	Yes	Cattle	South Africa	[51]
	PCR	16S rRNA	1470	Yes	Cattle, wildebeest, buffalo, waterbuck and eland	South Africa	[62]
	PCR	*groEL*	1482	Yes			
	PCR	*msp4*	800	Yes			
	qPCR	*msp1β*	95	No	African buffalo	Mozambique	[50]
	PCR	*msp5*	458	Yes	African buffalo	Mozambique	[50]
	PCR	*groEL*	520	Yes	African buffalo	Mozambique	[50]
	PCR	16S rRNA	502	Yes	African buffalo	Mozambique	[50]
	qPCR	*msp1β*	95	No	Cattle	Mozambique	[63]
	PCR	*msp4*	842	Yes	Cattle	Mozambique	[63]
	PCR	*msp5*	458	Yes	Cattle	Mozambique	[63]
	PCR	16S rRNA	492–498	No	African buffalo	Botswana	[8]
	RLB	*msp4*	420	Yes	Cattle	Tunisia	[64]
	PCR	*msp4*	344	No	Cattle	Tunisia	[43]
	PCR	*msp4*	852	Yes	Cattle	Tunisia	[43]
	PCR	16S rRNA	345	No	Cattle	Tunisia	[56]
	PCR	*msp4*	344	No	Cattle	Tunisia	[56]
	PCR	*lipA*	538	Yes	Cattle	Tunisia	[56]
	PCR	*sucB*	808	Yes	Cattle	Tunisia	[56]
	PCR	*OmpA*	711	Yes	Cattle	Tunisia	[56]
	PCR	*dnaA*	512	Yes	Cattle	Tunisia	[57]
	PCR/MSLT	*ftsZ*	575	Yes	Cattle	Tunisia	[57]
	PCR/MSLT	*groEL*	1025	Yes	Cattle	Tunisia	[57]
	PCR/MSLT	*lipA*	538	Yes	Cattle	Tunisia	[57]
	PCR/MSLT	*recA*	579	Yes	Cattle	Tunisia	[57]
	PCR/MSLT	*secY*	501	Yes	Cattle	Tunisia	[57]
	PCR/MSLT	*sucB*	508	Yes	Cattle	Tunisia	[57]
	qPCR	*msp1β*	95	No	Cattle	Tunisia	[65]
	PCR	*msp4*	852	Yes	Cattle	Tunisia	[65]
	PCR	16S rRNA	75	Yes	*Hy. excavatum*	Egypt	[48]
	qPCR	16S rRNA	345	Yes	*R. annulatus*	Egypt	[48]
	PCR	*msp5*	475	Yes	Tick	Egypt	[48]
	PCR	16S rRNA	–	No	Cattle	Egypt	[49]
	qPCR	*msp1β*	95	Yes	Cattle	Egypt	[46]
	PCR	*groEL*	866	Yes	Buffalo	Egypt	[55]
	RLB	16S rRNA	426		*Hy. excavatum* and *R. annulatus*	Egypt	[42]
	PCR	16S rRNA	492–498	Yes	Cattle	Egypt	[42]
	qPCR	*msp1β*	95	No	Cattle and buffalo	Egypt	[66]
	RLB	16S rRNA	460–500	Yes	Cattle and buffalo	Egypt	[66]
	PCR	*msp1α*	800–1000	Yes		Egypt	[66]
	qPCR	23S rRNA	169	Yes	Cattle	Algeria	[54]
	PCR	16S rRNA	475	No	Cattle	Algeria	[54]
	PCR	16S rRNA	492–498	Yes	Cattle	Sudan	[67]
	PCR	*msp4*	849	Yes	Cattle	Sudan	[67]
	PCR	*msp4*	849	Yes	Cattle	Nigeria	[13]
	PCR	*msp2*	1230	Yes	Cattle	Nigeria	[13]
	PCR	16S rRNA	421	No	Cattle	Nigeria	[52]
	RLB	16S rRNA	460–520	No	*R. microplus*	Nigeria	[21]
	qPCR	23S rRNA	169	Yes	*R. microplus*	Côte d’Ivoire	[23]
	PCR	*msp5*	500	Yes	Cattle	Côte d’Ivoire	[23]
	PCR	*msp4*	576	Yes	Cattle	Benin	[41]
	PCR	*groEL*	344	Yes	Cattle	Benin	[41]
	PCR	16S rRNA	885	Yes	Cattle	Benin	[41]
	PCR	*msp5*	460–520	No	Cattle	Cameroon	[29]
	PCR	*msp5*	547	Yes	Cattle	Tanzania	[68]
	nPCR	*msp5*	195	No	Cattle	Tanzania	[68]
	PCR	*msp5*	547	Yes	Cattle	Tanzania	[68]
	nPCR	*msp5*	195	No	Cattle	Tanzania	[69]
	nPCR	16S rRNA	195	Yes	Cattle	Kenya	[40]
	PCR-HRM	16S rRNA	300	No	Cattle	Kenya	[40]
	PCR	16S rRNA	1060	Yes	Cattle	Kenya	[16]
	nPCR	16S rRNA	1030	Yes	Cattle	Kenya	[16]
	PCR	16S rRNA	424	No	Cattle	Kenya	[16]
	PCR-HRM	16S rRNA	300	Yes	Cattle	Kenya	[53]
	nPCR	16SrRNA	1090	No	Cattle	Kenya	[45]
	PCR	*groEL*	1668	Yes	Cattle	Uganda	[44]
	PCR	*groEL*	580	Yes	Cattle	Uganda	[44]
*A. centrale*	PCR	*groEL*	522	Yes	Cattle	South Africa	[60]
	qPCR	*groEL*	77	No	Cattle, wildebeest, buffalo, waterbuck and eland	South Africa	[74]
	PCR	msp1aS	637–937	Yes	Cattle, wildebeest and buffalo	South Africa	[74]
	PCR	16S rRNA	1470	Yes	Cattle	South Africa	[62]
	PCR	*groEL*	1482	Yes	wildebeest, buffalo, waterbuck and eland cattle and African buffalo	South Africa	[62]
	PCR	*msp4*	800	Yes			
	PCR	*msp5*	351	Yes	Cattle	Mozambique	[50]
	RLB	16S rRNA	492–498	No	Cattle	Botswana	[8]
	PCR	16S rRNA	426	Yes	Cattle	Tunisia	[43]
	qPCR	*groEL*	77	No	Cattle	Tunisia	[65]
	PCR	16S rRNA	1433	No	Cattle	Tunisia	[65]
	nPCR	16S rRNA	426	Yes	Cattle	Tunisia	[65]
	qPCR	16S rRNA	400	Yes	Cattle	Egypt	[46]
	PCR	16S rRNA	476	Yes	Cattle	Algeria	[54]
	PCR	16S rRNA	492–498	No	Cattle	Sudan	[67]
	PCR	*msp4*	849	Yes	*Am. variegatum*	Sudan	[67]
	RLB	16S rRNA	460–520	No	*Am. variegatum*	Nigeria	[21]
	qPCR	23S rRNA	169	No	Cattle	Côte d’Ivoire	[23]
	PCR	23S rRNA	500	Yes	Cattle	Côte d’Ivoire	[23]
	PCR	16S rRNA	460–520	Yes	Cattle	Cameroon	[29]
	PCR-HRM	16S rRNA	300	No	Cattle	Kenya	[45]
	nPCR	16S rRNA	1090	Yes	Cattle	Kenya	[45]
*A. phagocytophilum*	PCR	*msp2*	334	Yes	Cattle	Tunisia	[64]
	PCR	16S rRNA	1433	No	*Hy. aegyptium*	Tunisia	[90]
	nPCR	16S rRNA	641	Yes	*Hy. aegyptium*	Tunisia	[90]
	PCR	16S rRNA	345	Yes	*R. rattus*	Tunisia	[90]
	qPCR	23S rRNA	169	No	Cattle	Algeria	[11]
	PCR	23S rRNA	649	Yes	Cattle	Algeria Algeria	[11]
	PCR	16S rRNA	345	Yes	Cattle		[11]
	PCR-RFLP	16S rRNA	925	No	Cattle	Algeria	[22]
	PCR	16S rRNA	345	Yes	Baboons and vervet monkeys	Zambia	[88]
	PCR	16S rRNA	1470	Yes	Dogs and rodents	South Africa	[14]
	PCR	16S rRNA	700	Yes	Rodents and human	South Africa	[14]
	PCR	*gltA*	956	Yes	Dogs and rodent	South Africa	[14]
	nPCR	*gltA*	422	Yes	Dogs	South Africa	[14]
	PCR	16S rRNA	205	Yes	*H. elliptica*	South Africa	[91]
	PCR	16S rRNA	478	Yes	Lions, wild cats and servals	Zimbabwe	[87]
	PCR	16S rRNA	500	Yes	Buffalo	Mozambique	[50]
	PCR	16S rRNA	345	Yes	Cattle	Angola	[24]
*A. phagocytophilum*-like	PCR	16S rRNA	1433	No	Cattle	Uganda	[44]
	PCR	16S rRNA	926	Yes	Cattle	Uganda	[44]
	PCR	16S rRNA	641	Yes	Cattle	Uganda	[44]
	PCR	16S rRNA	345	Yes	*R. rattus*	Tunisia	[91]
	PCR	16S rRNA	1433–1434	No	Cattle, sheep, and goats	Tunisia	[94]
	nPCR	16S rRNA	641–642	Yes			
	PCR-RFLP	16S rRNA	-	No	*R. turanicus* and *R. sanguineus*	Tunisia	[10]
	PCR	16S rRNA	1433	No	*R. turanicus*	Tunisia	[10]
	nPCR	16S rRNA	641	Yes	*R. turanicus*	Tunisia	[10]
	PCR	16S rRNA	1433	No	Sheep and goats	Tunisia	[94]
	PCR-RFLP	16S rRNA	641–642	Yes	Sheep and goats	Tunisia	[93]
	nPCR	*groEL*	573	No	Sheep and goats	Tunisia	[93]
	nPCR	*groEL*	1446	Yes	Sheep and goats	Tunisia	[93]
	nPCR	*groEL*	792	Yes	Sheep and goats	Tunisia	[93]
	nPCR	16S rRNA	641	Yes	Sheep and goats	Tunisia	[94]
*A. platys*	RLB	16S rRNA	492–498	No	*Hy. excavatum*	Egypt	[42]
	PCR	16S rRNA	426	Yes	and *R. annulatus*	Egypt	[42]
	PCR	16S rRNA	426	Yes	Cattle	Egypt	[42]
	PCR	*groEL*	855	Yes	Cattle	Egypt	[66]
	PCR	*groEL*	777–825	Yes	Cattle	Nigeria	[13]
	PCR	16S rRNA	466–506	Yes	Cattle	Nigeria	[13]
	RLB	16S rRNA	460–520	Yes	Cattle	Nigeria	[21]
	RLB	16S rRNA	460–520	Yes	Camel	Nigeria	[106]
	PCR	16S rRNA	345	No	Dogs	Cape Verde	[105]
	PCR	16S rRNA	678–679	No	Dogs	Cape Verde	[105]
	PCR	16S rRNA	349	Yes	Dogs and ticks	Côte d’Ivoire	[107]
	PCR	*rpoB*	492	Yes	Dogs	Senegal	[28]
	PCR	16S rRNA	460–520	Yes	Cattle	Cameroon	[29]
	PCR	16S rRNA	424	Yes	Cattle	Kenya	[53]
	PCR	16S rRNA	349	Yes	Dogs and ticks	Kenya	[108]
	PCR-HRM	16S rRNA	200–300	Yes	Dogs, ticks, cattle, goats and sheep	Kenya	[109]
	qPCR	23S rRNA	169	No	Cattle	Algeria	[11]
	PCR	23S rRNA	649	Yes	Cattle	Algeria	[11]
	PCR	16S rRNA	345	Yes	*R. sanguineus*	Algeria	[11]
	qPCR	16S rRNA	142	Yes	Dogs	Algeria	[15]
	qPCR	*groEL*	-		Dogs	Morocco	[102]
	PCR	16S rRNA	1470	Yes	Dogs	South Africa	[14]
	PCR	16S rRNA	700	Yes	*R. evertsi evertsi*	South Africa	[14]
	PCR-RLB	16S rRNA	500	Yes	Dogs	South Africa	[101]
	PCR	16S rRNA	800–1487	Yes	Dogs	Zambia	[18]
	nPCR	*gltA*	430–950	Yes	Buffalo	Zambia	[18]
	PCR	16S rRNA	500	No	Cattle	Mozambique	[50]
	PCR	16S rRNA	345	Yes	Dogs and *R. sanguineus*	Angola	[24]
	PCR	16S rRNA	345	Yes		Congo	[96]
	PCR	16S rRNA	678	Yes	Dogs and *R. sanguineus* s. l.	Congo	[96]
	PCR	*groEL*	840–1277	Yes		Congo	[96]
	PCR	*gltA*	1302	Yes	Dogs and *R. sanguineus* s. l.	Congo	[96]
*A. platys*-like	PCR	16S rRNA	926	Yes	Cattle	Egypt	[55]
	PCR	16S rRNA	426	Yes	Cattle	Egypt	[66]
	PCR	16S rRNA	734	Yes	*R. annulata*	Egypt	[48]
	PCR	16SrRNA	476	Yes	*R. annulata*	Algeria	[54]
	PCR	16S rRNA	1433	Yes	Camel	Tunisia	[103]
	hn-PCR	*gltA*	947	Yes	Goats and sheep	Tunisia	[111]
	hn-PCR	*groEL*	518	Yes		Tunisia	[111]
	hn-PCR-	*groEL*	515	Yes	Goats and	Tunisia	[114]
	RFLP				sheep		
	PCR	16S rRNA	345	Yes	Sheep, goats and cattle	Tunisia	[112]
	hn-PCR	*groEL*	515	Yes			
	hn-PCR				Camels and *Hy. dromedarii*		
	-RFLP	*groEL*	515	No		Tunisia	[112]
	PCR-HRM				Camels and *Hy. dromedarii*		
	PCR	16S rRNA	300	Yes		Kenya	[16]
	nPCR	16S rRNA	1060	No	Cattle	Kenya	[16]
	PCR-HRM	16S rRNA	1030	Yes	Cattle	Kenya	[16]
	nPCR	16S rRNA	300	No	Cattle	Kenya	[45]
	nPCR	16S rRNA	1090	Yes	Cattle	Kenya	[45]
	PCR	16S rRNA	925	Yes	Cattle	Kenya	[109]
					*R. decoloratus* and *Am. variegatum*		
	nPCR	16SrRNA	800	Yes	Cattle	Mozambique	[63]
	nPCR	*groEL*	1297	Yes	Cattle	Mozambique	[63]
	qPCR	16S rRNA	97	Yes	Dogs and *R. sanguineus*	Nigeria	[104]
*A. ovis*	PCR	*msp4*	347	Yes	Sheep	Egypt	[55]
	PCR	16S rRNA	476	Yes	Sheep, cattle, goats, *R. sanguineus* and *R. bursa*	Algeria	[54]
	qPCR	23S rRNA	280	No	Sheep, goats, *R. turanicus* and *R. bursa*	Algeria	[117]
	PCR	23S rRNA	649	Yes			
	PCR	16S rRNA	492–498	No	Cattle, sheep	Sudan	[67]
	PCR	*msp4*	849	Yes	and goats		
	PCR	16S rRNA	345	No	R. rattus	Tunisia	[90]
	PCR	*msp4*	852	Yes	*R. rattus*	Tunisia	[90]
	PCR	*msp4*	852	Yes	*R. turanicus* and *R. sanguineus*	Tunisia	[10]
	PCR	16S rRNA	522	Yes		Tunisia	[10]
	PCR	groEL	722	Yes	*R. turanicus* and *R. sanguineus* s.l.	Tunisia	[10]
	PCR	*msp4*	344	Yes	Sheep and goats	Tunisia	[9]
	PCR	*msp4*	852	Yes	Sheep and goats	Tunisia	[9]
	PCR	*msp4*	344	No	Sheep	Tunisia	[94]
	LAMP	*msp4*	-	No	Sheep	Tunisia	[94]
	PCR	*msp4*	852	Yes	Sheep	Tunisia	[94]
	PCR	16S rRNA	374	No	Sheep	Tunisia	[124]
	PCR	*msp4*	852	Yes	Sheep	Tunisia	[124]
	PCR	*gltA*	760	Yes	Goats, sheep and camel	Tunisia	[125]
	PCR	*groEL*	722	Yes	Goats and sheep	Tunisia	[125]
	PCR	msp1a	500–750	Yes	Goats, sheep and *R. turanicus*	Tunisia	[125]
	PCR	*msp4*	374	No	Camel	Tunisia	[123]
	PCR	*msp4*	852	Yes	Camel	Tunisia	[123]
	PCR	groEL	722	Yes	*R. evertsi* and *R. appendiculatus*	Tunisia	[123]
	PCR	*msp4*	344	No	Sheep and goats	Tunisia	[94]
	PCR	*msp4*	852	Yes	Sheep and goats	Tunisia	[94]
	PCR	16S rRNA	451	Yes	*R. evertsi* and *R. decoloratus*	Ethiopia	[120]
	PCR-HRM	16S rRNA	112–200	No	Sheep and ticks	Kenya	[17]
	PCR	16S rRNA	300	Yes	Cattle, sheep, goats and ticks	Kenya	[119]
	PCR	*msp4*	347	Yes		Kenya	[119]
	PCR-HRM	16S rRNA	300	No	Sheep and goats	Kenya	[26]
	PCR	16S rRNA	1030	Yes	Sheep and goats	Kenya	[26]
	PCR-HRM	16S rRNA	200	No	*Am. hebraeum*	Kenya	[109]
	PCR	16S rRNA	330	Yes	*Am. hebraeum*	Kenya	[109]
	PCR	16S rRNA	430	No	*Am. hebraeum*	Uganda	[126]
	PCR	*msp4*	347	No	*Am. hebraeum*	Uganda	[126]
	PCR	*msp4*	347	No	Goats	South Africa	[121]
	PCR	16S rRNA	932	No	Goats	South Africa	[127]
	nPCR	16S rRNA	546	Yes	Goats	South Africa	[127]
	PCR	*msp4*	850	Yes	Sheep	Botswana	[122]
	PCR	*msp4*	92	No	Sheep	Botswana	[122]
	PCR	*rpoB*	483	Yes	Sheep	Senegal	[28]
*A. bovis*	PCR	16S rRNA	551	Yes	Cattle	Tunisia	[43]
	PCR	16S rRNA	1433	No	Sheep and goats	Tunisia	[9]
	PCR	16S rRNA	551	Yes	Sheep and goats	Tunisia	[9]
	PCR	16S rRNA	551	Yes	*Hy. dromedarii*	Tunisia	[130]
	PCR	16S rRNA	1433	No	Sheep and goats	Tunisia	[131]
	nPCR	16S rRNA	551	Yes	Sheep and goats	Tunisia	[131]
	PCR	16S rRNA	1433	No	Cattle	Tunisia	[65]
	nPCR	16S rRNA	551	Yes	Cattle	Tunisia	[65]
	PCR-HRM	16S rRNA	300	Yes	Cattle	Kenya	[16]
	PCR	16S rRNA	1060	No	Cattle	Kenya	[16]
	nPCR	16S rRNA	1030	Yes	Cattle	Kenya	[16]
	PCR	16S rRNA	424	Yes	Cattle	Kenya	[53]
	PCR-HRM	16S rRNA	112–200	No	*R. appendiculatus*	Kenya	[17]
	PCR	16S rRNA	300	Yes	Cattle, sheep, goats and ticks	Kenya	[109]
	PCR-HRM	16S rRNA	200	No			
	PCR	16S rRNA	330	Yes	*R. praetextatus*	Tanzania	[132]
	PCR	16S rRNA	452	Yes	*R. evertsi*	Tanzania	[132]
	PCR	16S rRNA	932	No	*R. evertsi*	South Africa	[133]
	nPCR	16S rRNA	546	Yes	*Rhipicephalus* sp.	South Africa	[133]
	PCR	16S rRNA	247	Yes	*E. myurus*	South Africa	[134]
	PCR	16S rRNA	345	Yes	Cats	Angola	[135]
	PCR	16S rRNA	123	No	Cats	Angola	[135]
	PCR	16S rRNA	345	Yes	Cats	Angola	[135]
*A. capra*	PCR	16S rRNA	345	Yes	Cattle	Angola	[24]
	PCR	16S rRNA	345	No	Dogs	South Africa	[19]
*Anaplasma* sp. SA dog	PCR	16S rRNA	1389	Yes	Dogs	South Africa	[19]
	PCR	*gltA*	431	Yes	Dogs	South Africa	[19]
	RLB	16S rRNA	492–498	No	Dogs	South Africa	[20]
	PCR	16S rRNA	492–498	Yes	Dogs	South Africa	[20]
	PCR	16S rRNA	1470	Yes	Dogs and *R. sanguineus*	South Africa	[14]
	PCR	16S rRNA	700	Yes			
	PCR	*gltA*	956	Yes	Dogs	South Africa	[14]
	nPCR	*gltA*	422	Yes	Dogs	South Africa	[14]
	PCR	16S rRNA	250	No	Dogs	Zambia	[18]
*Anaplasma* sp. ZAM dog	PCR	16S rRNA	800–1470	Yes	Dogs	Zambia	[18]
	PCR	*gltA*	430–950	Yes	Dogs	Zambia	[18]
*Ca.* Anaplasma boleense	PCR	16S rRNA	1470	Yes	Cattle	South Africa	[14]
	PCR	*groEL*	792	Yes	Cattle and sheep	Senegal	[142]
*Anaplasma* sp. Mymensingh	PCR	16S rRNA	1470	Yes	Cattle	South Africa	[14]
*Anaplasma* sp. Omatjenne	PCR	16S rRNA	1449	Yes	Boer goats	South Africa	[6]
	RLB	16S rRNA	460–520	No	Cattle	Nigeria	[21]
	PCR-RFLP	16S rRNA	925	No	Cattle	Ethiopia, Côte d’Ivoire, Zambia, Rwanda and Morocco	[22]
*Ca.* Anaplasma ivoriensis	qPCR	23S rRNA	169	No	*Am. variegatum* and *R. microplus*	Côte d’Ivoire	[23]
	PCR	23S rRNA	500	Yes			
*Ca.* Anaplasma turritanum	nPCR	*groEL*	573	Yes	Sheep and goats	Senegal	[142]
	nPCR	*gltA*	947	Yes			
*Ca.* Anaplasma cinensis	nPCR	*groEL*	573	Yes	Cattle	Senegal	[142]
	nPCR	*gltA*	660	Yes	Cattle	Senegal	[142]
*Ca.* Anaplasma sphenisci	PCR	16S rRNA	927	Yes	African penguin	South Africa	[31]
	nPCR	*groEL*	939	Yes			
*Ca.* Anaplasma camelii	nPCR	16S rRNA	426	Yes	Camel	Nigeria	[27]
	PCR-HRM	16S rRNA	300	No	Camel, *Hyalomma*, *Amblyomma* and *Rhipicephalus* spp.	Kenya	[26]
	PCR	16S rRNA	1030	Yes			
*Ca.* Anaplasma africae	PCR	*rpoB*	568	Yes	Sheep, goats and cattle	Senegal	[28]
*Anaplasma* sp. Hadesa	PCR	16S rRNA	460–520	Yes	Cattle	Cameroon	[29]
	PCR-RLB	16S rRNA	460–500	No	Cattle	Ethiopia	[30]
	PCR	16S rRNA	1438	Yes	Cattle	Ethiopia	[30]
*Anaplasma* sp. Saso	PCR-RLB	16S rRNA	460–500	No	Cattle	Ethiopia	[30]
	PCR	16S rRNA	1438	Yes	Cattle	Ethiopia	[30]
*Anaplasma* sp. Dedessa	PCR-RLB	16S rRNA	460–500	No	Cattle	Ethiopia	[30]
	PCR	16S rRNA	1438	Yes	Cattle	Ethiopia	[30]
*Anaplasma* sp. Lambwe	PCR-HRM	16S rRNA	300	Yes	Cattle	Kenya	[16]
	PCR	16S rRNA	1060	No	Cattle	Kenya	[16]
	nPCR	16S rRNA	1030	Yes	Cattle	Kenya	[16]
*Anaplasma* sp.	PCR	16S rRNA	424	Yes	Cattle	Kenya	[53]
	PCR	16S rRNA	335-430	Yes	Sheep	Kenya	[119]
	PCR	16S rRNA	424	Yes	Cattle	Kenya	[53]
	PCR	16S rRNA	925	Yes	*R. pravus*	Kenya	[109]
	PCR	16S rRNA	925	Yes	*R. decoloratus*	Ethiopia	[147]
	PCR	16S rRNA	257	Yes	*Am. cohaerens*	Ethiopia	[147]
	qPCR	23S rRNA	169	No	Cattle	Algeria	[11]
	PCR	23S rRNA	649	Yes	Cattle	Algeria	[11]
	PCR	16S rRNA	345	Yes	Cattle	Algeria	[11]
	PCR	16S rRNA	1433	Yes	*Hy. dromedarii*	Tunisia	[131]
	PCR	16S rRNA	426	Yes	Buffalo	Egypt	[66]
	PCR	16S rRNA	250	Yes	*Rhipicephalus* and *Amblyomma* spp.	South Africa	[143]
	PCR	16S rRNA	250	Yes	*R. sanguineus*, *H. elliptica* and *Am. hebraeum*	South Africa	[144]
	PCR	16S rRNA	250	Yes	*Rhipicephalus*, *Hyalomma* and *Otobius* spp.	Lesotho	[145]
	PCR	16S rRNA	250	Yes	Brown and spotted hyenas	Namibia and Tanzania	[148]
	PCR	16S rRNA	400-600	No	*Am. gemma*	Tanzania	[149]
	PCR	16S rRNA	345	No	*Ar. walkerae* and *O. moubata*	Zambia	[146]
	PCR	16S rRNA	1300	Yes	*Ar. walkerae* and *O. moubata*	Zambia	[146]
	nPCR	*groEL*	1297	Yes	*I. aulacodi*	Ghana	[25]
	PCR	16S rRNA	345	No	*I. aulacodi*	Ghana	[25]
	nPCR	*gltA*	1236	Yes	*I. aulacodi*	Ghana	[25]
	nPCR	*groEL*	1320	Yes	*I. aulacodi*	Ghana	[25]
	qPCR	23S rRNA	190	No	Rodents	Gabon	[150]
	nPCR	23S rRNA	650	Yes	Rodents	Gabon	[150]

* Abbreviations: nPCR: nested PCR; hn-PCR: heminested PCR; qPCR: quantitative real-time PCR, PCR-RLB: PCR followed by reverse line blot hybridization assay; PCR-RFLP: PCR followed by restriction fragment length polymorphism assay; PCR-HRM: PCR followed by high-resolution melting analysis; LAMP: loop-mediated isothermal amplification assay; PCR/MLST: PCR and multilocus sequence typing.

## 3. Anaplasmosis Control in Africa

In general, anaplasmosis control measures vary with the geographic locality, and are dependent on the accessibility, affordability, and the practicality of the application [151]. In the past, in regions where the disease is not endemic, anaplasmosis control has been largely implemented by the preservation of *A. marginale*-free herds. This was done to prevent the introduction of *Anaplasma*-infected carrier animals that could serve as portals of infection to these nonendemic areas [151].

### 3.1. Control of Anaplasmosis by Vaccination

Control of bovine anaplasmosis caused by *A. marginale* includes the use of a live *A. centrale* vaccine developed by Arnold Theiler over a century ago in South Africa [152,153]. This vaccine has been widely utilized in many regions of the world and is effective in preventing clinical disease after infection caused by field strains of *A. marginale* [4,73,154]. However, it has the limitations of offering only partial protection when challenged by diverse strains of *A. marginale* and is likely to introduce new strains of infection in regions where *A. marginale* is nonendemic; thus, it is not used in such countries as the United States [155]. Other vaccines that have been developed to prevent bovine anaplasmosis include inactivated, cultured or killed *A. marginale* vaccines [73,156,157,158]. These vaccines have the drawbacks of being partially effective, not suitable for large-scale production, and the occurrence of associated safety concerns that have been linked to their use [159]. Subunit recombinant vaccines have been advocated to be a practical and viable option for producing large-scale uniform vaccine stocks [160,161], with experimental studies showing that outer membrane protein (OMP) of *A. marginale* can induce protection by limiting the severity of clinical infections in vaccinated animals [162,163]. Analysis of OmpA protein sequences obtained from Tunisian cattle identified putative immunodominant epitopes of B and T cells that showed high conservation in Tunisian isolates and in other isolates around the world [56]. The study speculated that minor intraspecific differences should not influence the possible cross-protective ability of antibody-mediated and cellular immune responses against various *A. marginale* strains worldwide [56]. In South Africa, a study identified five recombinant *A. marginale* OMPs from strains of *A. marginale* in the country that were suggested to be interesting vaccine candidates for use in novel global vaccine cocktails against *A. marginale* [155].

### 3.2. Tick Control as a Mechanism to Control Anaplasmosis

Prevention of anaplasmosis in domestic animals has been largely based on controlling tick infestation through the use of acaricides via dipping and the utilization of pour-on or spot-on administration of organophosphates, formamidines, synthetic pyrethroids, and macrocyclic lactones [164]. However, the continuous and improper use of acaricides to control ticks has led to the increased incidence of acaricide resistant ticks [165] and the contamination of meat and milk products and the environment [166]. In Africa, to control tick infestations, the use of lower cost, nontoxic and environmentally friendly plant extracts as an alternative to chemical acaricides has been reported to be effective against *R. decoloratus* [167,168], *R. pulchellus* [169], *R. microplus* [170], *R. appendiculatus* [171,172], *Hy. rufipes* [173,174,175,176], and *Hy. anatolicum* [177].

Tick vaccines such as the commercially available cement antigen vaccines Bm86-based TickGARD™ Plus and Gavac^®^ have been developed and tested [178]. These vaccines cause an antibody-mediated response in the tick that causes the rupture of the midgut, reduced reproduction and tick death [179,180]. A vaccine that silences subolesin (SUB) expression has also been reported [181]. Subolesin is a tick protective antigen that has been associated with modulating the activities of tick feeding, reproduction and blood-meal digestion [181]. Tick vaccines have the advantages of being cheaper to produce and impacting less harm to the environment when compared to acaricide use [182].

In Uganda, a study used the orthologue of the gut protein Bm86 in *R. appendiculatus* (Ra86) in rabbit immunization trials against all life stages of *R. appendiculatus* and found 23.1% mortality in the adult ticks compared to 1.9% in the control group. However, the vaccine was ineffective against the larval and nymphal stages of the tick [183]. Additionally, SUB-based vaccines were tested against *R. appendiculatus*, *R. decoloratus* and *Am. variegatum* that affect the production of common cattle breeds in Uganda, showing that *R. appendiculatus* SUB was more cross-protective than the other tested antigens and was a useful tool for subsequent vaccine-based research on the control of cattle ticks in the country [184]. In Kenya, the commercial TickGARD™ Plus was tested against *R. appendiculatus* infesting *Bos indicus* calves [185]. The vaccine showed limited protection against the ticks, but caused a significant decrease in the mean engorged weight of *R. decoloratus* and reduced the egg mass laid by surviving adult female ticks [185]. In Nigeria, molecular characterization of the Bm86 gene homologues in *Hyalomma* spp., *R. annulatus* and *R. decoloratus* was undertaken towards the development of an anti-tick vaccine [186]. The study found a 100% homology in *Rhipicephalus* spp., but the sequence was divergent in *Hyalomma* spp. [186]. Phylogenetic analysis indicated a 3–8% sequence variation between the hosts and other nucleotide sequences from the USA, Australia, Israel and South Africa, suggesting that limited cross-protection will be provided by the Bm86 gene homologues [186].

In Tunisia, a study amplified, cloned and sequenced transcripts of the orthologues of the Bm86 gene in *Hyalomma scupense*, the tick vector implicated in causing the highest rates of infestation in livestock in North Africa [187]. Sequence analysis recorded an interspecific diversity of 35%-40% between Hd86, which is the orthologue of Bm86 in *Hy. scupense* and Bm86 proteins [187]. A minimal intraspecific diversity of 1.7% was, however, observed between the Hd86 vaccine candidate (Hd86-A1) and other homologues from *Hy. scupense* [187]. The study concluded by recommending the importance of a comparative study to examine the effects of the recombinant Bm86 and Hd86 vaccines against *Hy. scupense* [187]. In a subsequent study, vaccine trials in cattle using the Bm86 and Hd86 vaccines were performed against juvenile and adult *Hy. scupense* and adult *Hy. excavatum* [188]. The study found a 59.19% reduction in the number of *Hy. scupense* nymphs that became engorged on cattle that were vaccinated with Hd86 [188]. The Bm86 and Hd86 vaccinations, however, did not show any efficacy on reducing infestations by adult *Hy. scupense* and *Hy. excavatum* [188]. Follow-up research characterized Hd86 antigen mRNA levels in different life stages of *Hy. scupense* using qPCR and found a significant variation in the expression profile of Hd86 between different life stages of the tick [189]. The number of transcripts during the course of feeding and immediately after the molting phase in adults were markedly reduced in juvenile ticks, while the reverse was observed in adult ticks after feeding [189]. The authors postulated that the differences in Hd86 expression profiles in juvenile and adult *Hy. scupense* might explain the conflict in the efficacy of the Hd86 vaccine in the two life stages documented in the previous study [188,189].

Additional research in Tunisia amplified, cloned and sequenced transcripts of the Bm86 protein orthologue in *Hy. marginatum marginatum* (Hmm), *Hy. excavatum* (He) and *Hy. dromedarii* (Hdr) [190]. Analysis of eight full epidermal growth factor (EGF)-like regions and two partial EGF-like regions in Hmm, Hd and Hdr with the vaccine candidate from *Hy. scupense* (Hd86-A1) revealed a pronounced conservation of 87–91% similarity with this orthologue of Bm86 [190]. On the other hand, similarity indices of amino acid sequences of Bm86 orthologues of Hmm, Hd and Hdr (Hmm86, He86 and Hdr86) with the Bm86 protein from *R. microplus* only ranged between 60% and 66% [190]. The results from the study suggested the Hd86-A1 vaccine candidate was better suited for *Hyalomma* species than the commercially available Bm86-based vaccines [190]. Similar research in the country characterized Bm86 orthologues in *Hy. excavatum*, *Hy. anatolicum*, *Hy. marginatum marginatum* and *Hy. scupense* ticks [191]. Analysis of obtained amino acid sequences showed a high diversity of 33–34% in Bm86 and *Hy. excavatum* orthologues (He86-A1/A2/A3), implying a reduction in the efficacy of the Bm86-based commercial and experimental vaccines [191]. A limited 10.2% amino acid diversity between Hd86-A1 used in the experimental vaccine against *Hy. scupense* and He86-A1/A2/A3 was in agreement with the previous study that indicated that Hd86-A1 vaccine candidate might be a better vaccine target against the *Hy. excavatum* tick in comparison to the other Bm86 vaccines [190].

## 4. Concluding Remarks and Future Direction

The 16S rRNA gene has been the most widely utilized genetic marker in the characterization of *Anaplasma* species in Africa. Classification of *Anaplasma* to species level has, however, been shown to be difficult based on 16S rRNA gene sequences alone, as the gene is very similar across species. Studies that utilize the characterization of other *Anaplasma* full-length genes, such as *gltA*, 23S rRNA, *groEL*, *drhm*, *vir*, and *ankA* loci in conjunction with the 16S rRNA gene should be undertaken to clearly differentiate and designate species. The use of MLST and next-generation sequencing (NGS) would also help to elucidate the genetic diversity of *Anaplasma* spp. in Africa. There is currently a paucity of information on the detection of *Anaplasma* spp. in argasid and avian ticks in Africa. Future research of *Anaplasmataceae* in argasid and avian ticks on the continent will advance knowledge on the evolution and epidemiology of these organisms in these understudied vectors and hosts.

Due to their obligate intracellular nature, *Anaplasma* species are difficult to culture in the laboratory, as current techniques necessitate the use of mammalian and arthropod cells for their replication. Research on the development of an axenic media to culture *Anaplasma*—a feat achieved with another intracellular organism, *Coxiella burnetii* will facilitate the production of high-quality genetic material, which is essential for whole-genome sequencing.

In conclusion, the generation of whole-genome *Anaplasma* sequences from various animal hosts, ticks and geographical regions on the continent is essential in delineating the diversity of the *Anaplasma* genus in Africa. Whole-genome sequencing studies will unveil the entire genetic diversity of *Anaplasma* spp. on the continent and subsequently ease the development of other whole-genome typing methodologies, such as single-nucleotide polymorphism applications or whole-genome MLST.

## Data Availability

Not applicable.
